# Treatment with Vitamin D/MOG Association Suppresses Experimental Autoimmune Encephalomyelitis

**DOI:** 10.1371/journal.pone.0125836

**Published:** 2015-05-12

**Authors:** Fernanda Chiuso-Minicucci, Larissa Lumi Watanabe Ishikawa, Luiza Ayumi Nishiyama Mimura, Thais Fernanda de Campos Fraga-Silva, Thais Graziela Donegá França, Sofia Fernanda Gonçalves Zorzella-Pezavento, Camila Marques, Maura Rosane Valerio Ikoma, Alexandrina Sartori

**Affiliations:** 1 Department of Microbiology and Immunology, Biosciences Institute, Universidade Estadual Paulista (UNESP), Botucatu, São Paulo, Brazil; 2 Laboratório de Citometria de Fluxo—Fundação Dr. Amaral Carvalho, Jaú, São Paulo, Brazil; University of Texas at San Antonio, UNITED STATES

## Abstract

Experimental autoimmune encephalomyelitis (EAE) is an animal model to study multiple sclerosis (MS). Considering the tolerogenic effects of active vitamin D, we evaluated the therapeutic effect of myelin oligodendrocyte glycoprotein (MOG) associated with active vitamin D in EAE development. EAE was induced in female C57BL/6 mice by immunization with MOG emulsified with Complete Freund’s Adjuvant plus *Mycobacterium tuberculosis*. Animals also received two intraperitoneal doses of *Bordetella pertussis* toxin. One day after immunization, mice were treated with 0,1μg of 1α,25-dihydroxyvitamin D3 (1,25(OH)_2_D_3_) every other day during 15 days (on days 1, 3, 5, 7, 9, 11, 13 and 15). MOG (150μg) was co-administered on days 3 and 11. The administration of 1,25(OH) _2_D_3_ or MOG determined significant reduction in EAE incidence and in clinical scores. When MOG was associated with 1,25(OH) _2_D_3_ the animals did not develop EAE. Spleen and central nervous system (CNS) cell cultures from this group produced less IL-6 and IL-17 upon stimulation with MOG in comparison to the EAE control group. In addition, this treatment inhibited dendritic cells maturation in the spleen and reduced inflammatory infiltration in the CNS. The association of MOG with 1,25(OH) _2_D_3_ was able to control EAE development.

## Introduction

Multiple sclerosis (MS) is a demyelinating disease mediated by an immune response against self-antigens from the central nervous system (CNS). This immune response triggers an initial inflammation in brain and spinal cord that is then followed by demyelination, axonal damage and scar formation [1]. Experimental autoimmune encephalomyelitis (EAE) is an induced inflammatory demyelinating disease of the CNS that is largely employed as a model to study MS [2,3]. The pathogenic immune response observed in MS and EAE CNS is mainly mediated by Th1 and Th17 cells whose main signature cytokines are IFN-γ and IL-17, respectively [4–6]. Current MS treatment consists in general suppression of the immune response which can increase susceptibility to infections. Therefore, an antigen-specific tolerogenic strategy is highly desirable to treat this disease. Administration of antigens derived from myelin by intravenous, transdermal and oral routes protected animals from EAE development [7–9]. The transdermal application of myelin peptides in MS patients was also effective, being able to reduce magnetic resonance imaging and clinical parameters of disease activity [10]. Many of these strategies induced tolerogenic dendritic cells (DCs) whose tolerogenic properties can also be achieved by the use of pharmacological agents as corticosteroids, rapamycin, mycophenolate mofetil, prostaglandin E2 and vitamin D [11,12].

The active form of vitamin D, 1α,25-dihydroxyvitamin D3 (1,25(OH)_2_D_3_), is mainly obtained after sunlight exposure. 1,25(OH)_2_D_3_ has an important role in bone metabolism, in calcium and phosphorus homeostasis and also plays a regulatory role in the immune system [13]. Its activity is mediated by the vitamin D receptor which is expressed in many cell types including immune cells such as monocytes, macrophages, DCs and activated B and T lymphocytes [14]. In the innate immunity, it enhances the antimicrobial properties of monocytes and macrophages [13]. The regulatory role of 1,25(OH)_2_D_3_ in the adaptive immunity has been mainly attributed to its effects on DCs. *In vitro* exposure to 1,25(OH)_2_D_3_ inhibits DC maturation by reducing expression of markers such as CD1a, MHC II, CD40, CD80 and CD86 [15]. In addition, 1,25(OH)_2_D_3_ modulates cytokine production by DCs, inhibiting, for example, the production of IL-12 and IL-23 that induce Th1 and Th17 differentiation, respectively. Classically, it has been described that 1,25(OH)_2_D_3_ also increases the secretion of IL-10, a cytokine with very well established anti-inflammatory properties [16]. The effects of 1,25(OH)_2_D_3_ on T cells include inhibition of their proliferation, of IL-2 and IFN-γ production [17] and also of Th17 cells differentiation [18]. Besides, *in vitro* and *in vivo* studies show that 1,25(OH)_2_D_3_ usually induces Foxp3+ regulatory T cells [19]. The beneficial role of 1,25(OH)_2_D_3_ in experimental models of autoimmune diseases, as experimental autoimmune uveitis, EAE and diabetes have been clearly demonstrated [20–24]. A great deal of attention has been dedicated to reveal the detailed mechanism of 1,25(OH)_2_D_3_ over the immune system. Chang et al., 2010 [22], for example, showed that the beneficial effect of this substance in EAE is mediated throughout inhibition of differentiation and also migration of Th17 cells to the CNS. The contribution of tolerogenic DCs induced by 1,25(OH)_2_D_3_ to control autoimmunity was clearly showed by Farias et al., 2013 [23]. These authors demonstrated that both, *in vivo* administration of 1,25(OH)_2_D_3_ and transfer of 1,25(OH)_2_D_3_-induced IDO(+) immature DCs, significantly reduced EAE severity.

The aim of this study was to evaluate the efficacy of a precocious treatment with MOG/1,25(OH)_2_D_3_ association on EAE development.

## Materials and Methods

### Ethics statement

All animal studies and procedures were approved by local Ethics Committee for animal experimentation (CEEA), Universidade Estadual Paulista (protocol number 314). The animals were anesthetized by intraperitoneal injection of a solution containing ketamine and xylasin.

### Animals

Female C57BL/6 mice with 6–9 weeks old were purchased from University of São Paulo (USP, Ribeirão Preto, SP, Brazil). The animals received sterilized food and water *ad libitum*.

### EAE induction

MOG35–55 peptide (MEVGWYRSPFSRVVHLYRNGK) was synthesized by Genemed Synthesis Inc. (San Antonio, Texas, USA). Mice were immunized subcutaneously with 150 μg of MOG35-55 peptide emulsified in Complete Freund’s Adjuvant containing 4mg/mL of *Mycobacterium tuberculosis*. Mice also received two intraperitoneal doses, 0 and 48 h after immunization, of 250 ng of *Bordetella pertussis* toxin (Sigma Aldrich, St. Louis, MO, USA). Clinical assessment of EAE was daily performed according to the following criteria: 0—no disease, 1—limp tail, 2—weak hind legs, 3—partially paralyzed hind legs, 4—complete hind leg paralysis, and 5—complete paralysis/death.

### Treatment with 1,25(OH)_2_D_3_, MOG or 1,25(OH)_2_D_3_ associated with MOG

One day after EAE induction, mice were treated, by intraperitoneal route, with 0,1 μg of 1α,25-dihydroxyvitamin D3 (1,25(OH)_2_D_3_) from Sigma Aldrich, every other day during 15 days (on days 1, 3, 5, 7, 9, 11, 13 and 15); MOG (150μg) was co-administered, also by this route, on days 3 and 11.

### CNS-mononuclear cells isolation

Ten and eighteen days after EAE induction, mice were anesthetized with ketamine/xylazine and perfused with 10 mL of saline solution. Mononuclear cells from CNS were isolated as previously described [25]. Briefly, brain and spinal cord were entirely collected, macerated and digested with collagenase D (2.5mg/mL, Roche Diagnostics, Indianapolis, IN, USA) in 4mL of RPMI (Sigma Aldrich, St. Louis, MO, USA) at 37°C for 45 min. Suspensions were then washed in RPMI and centrifuged at 450 ×g for 15min at 4°C. Cells were resuspended in percoll (GE Healthcare, Uppsala, Sweden) 37% and laid over percoll 70%. The material was centrifuged at 950 ×g for 20 min with centrifuge breaks turned off and the mononuclear cell ring was collected, washed in RPMI, and centrifuged at 450 ×g for 10 min. Cells were then resuspended in complete RPMI medium, counted, and analyzed. To get enough cells to perform the experiments we used pools from 3 animals.

### Cell culture conditions and cytokine quantification

Eighteen days after EAE induction, spleen and CNS-isolated cells, whose viability was over 95%, were adjusted to 5 x 10^6^ cells/mL and 2 x 10^5^ cells/mL, respectively and cultured in RPMI medium containing 10% of fetal calf serum and 2 mM of glutamine. Spleen and CNS cultures were stimulated with 20 μg/mL and 50 μg/mL of MOG, respectively. Cytokine levels were evaluated 48h later by enzyme-linked immunosorbent assay (ELISA) using IFN-γ and IL-10 BD OptEIA Sets (BD Biosciences, San Jose, CA, USA) and IL-6 and IL-17 Duosets (R&D Systems, Minneapolis, MN, USA). The assays were performed according to the manufacturer’s instructions.

### Flow cytometry

Spleen and lymph nodes (popliteal + inguinal) cells were collected and the red blood cells were lysed with buffer containing NH_4_Cl. For regulatory T cells analysis, spleen, lymph nodes and CNS-isolated cells were incubated with 0.5 μg of FITC labeled anti-mouse CD4 (clone GK1.5) and 0.25 μg of APC labeled anti-mouse CD25 (clone PC61.5) for 20 min at room temperature. Intracellular Foxp3 transcription factor was detected using Foxp3 PE Staining Set (eBiosciences, San Diego, CA, USA) according to manufacturer's instructions. For DCs analysis, splenocytes were incubated with 0.25 μg of FITC labeled anti-mouse CD11c (clone N418), 0.03 μg of APC labeled anti-mouse MHCII (clone M5/114.15.2) and 0.125 μg of PE labeled anti-mouse CD86 (clone GL1) for 30 min at 4°C. The cells were then washed, resuspended in flow cytometry buffer and fixed in paraformaldehyde 1%. The cells were analyzed by flow cytometry using the FACSCanto II (BD Biosciences, San Jose, CA, USA). Regulatory T cells and DCs analyses were performed by FlowJo software (TreeStar, Ashland, OR, EUA) and Infinicyt software (Cytognos, Salamanca, Spain), respectively. These experiments were performed 18 days after EAE induction except Foxp3+ regulatory T cells (Treg) evaluation in the spleen and lymph nodes that was also assessed at the preclinical phase.

### Evaluation of inflammatory infiltrates in the CNS

The histological analysis was performed during EAE acute phase, that is, 18 days after disease induction. After euthanasia, lumbar spinal cord samples were removed and fixed in 10% formaldehyde. Paraffin slides with 5 μm were stained with hematoxylin and eosin (H&E) and analyzed with a Nikon microscope.

### Calcium and phosphorus levels

Calcium and phosphorus serum levels were analyzed using Cálcio Arsenazo III commercial kit (Bioclin, Minas Gerais, Brazil) and Quimifos-Fósforo commercial kit (Ebram, São Paulo, Brazil). The assays were performed according to the manufacturer’s instruction.

### Statistical analysis

Results were expressed as mean ± standard deviation or with median and interquartile (25–75%) ranges. Comparisons between groups were made by t-test or one way ANOVA followed by Tukey’s test for parametric variables and by Kruskal-Wallis followed by Dunn’s test for non-parametric variables. Chi-square was performed for EAE incidence. Statistical analysis was accomplished with SigmaPlot software version 12.0 (Systat Software Inc., San Jose, CA, USA) and p<0.05 was considered significant. See S1 File for full dataset.

## Results

### MOG/1,25(OH)_2_D_3_ association inhibits EAE development

**Fig 1:**
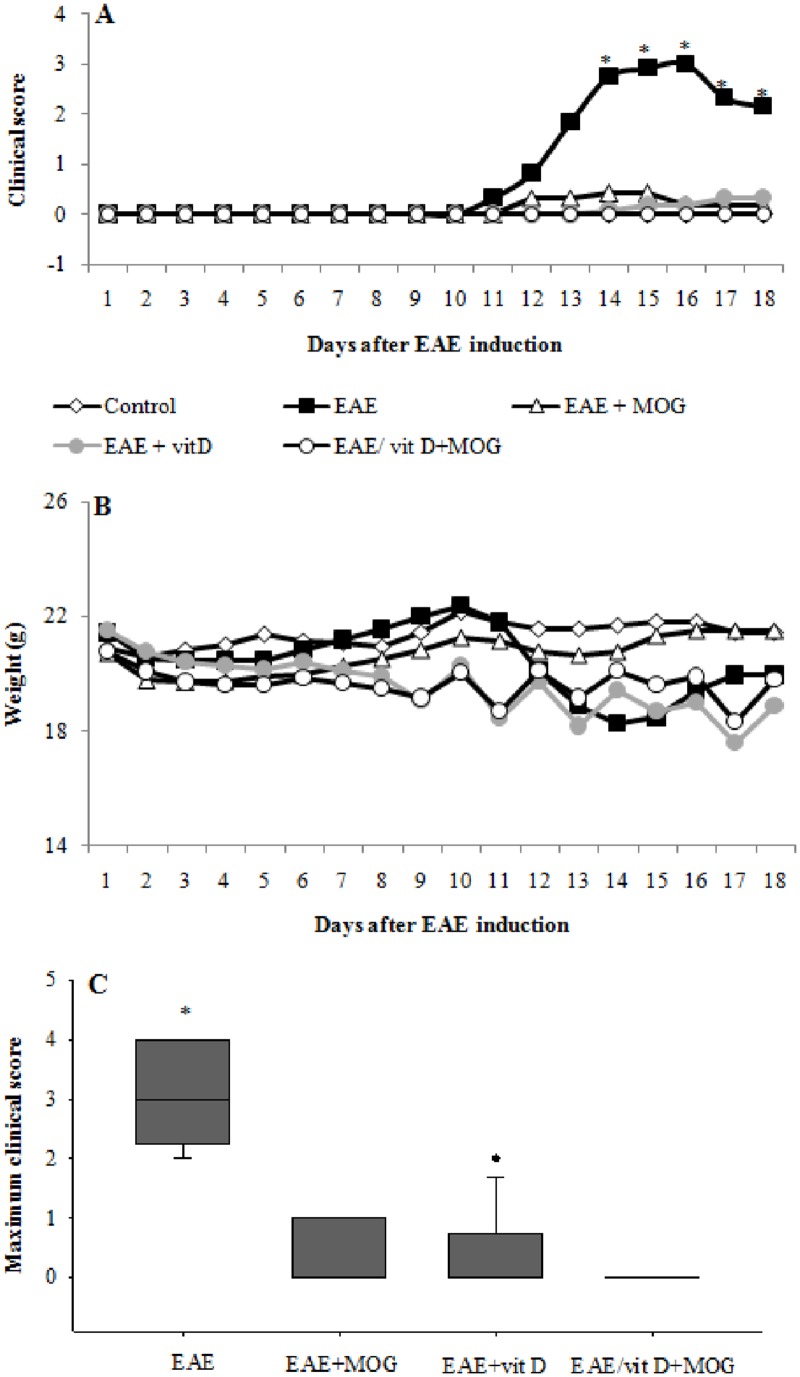
Effect of treatment with 1,25(OH)_2_D_3_+MOG association on EAE development. Kinetics of clinical scores (**A**), maximum clinical score (**B**) and weight variation (**C**). Comparisons between groups were made by one way ANOVA followed by Tukey’s test for parametric variables (**A** and **C**) and by Kruskal-Wallis followed by Dunn’s test for non-parametric variables (**B**). Results were expressed as mean or medians (25–75% ranges) of 12 animals per group. * p<0.05 compared with EAE+MOG, EAE+vitD and EAE/vitD+MOG groups. Data are representative of two independent experiments.

A 100% disease incidence was observed in the positive control group. These animals also presented very typical signs of disease characterized by elevated clinical score and accentuated body weight loss. On the other hand, groups treated with MOG, 1,25(OH)_2_D_3_ or 1,25(OH)_2_D_3_+MOG showed a decreased incidence and also a much less severe clinical disease. Distinctly from MOG and 1,25(OH)_2_D_3_ treated groups that showed a residual disease, the group treated with 1,25(OH)_2_D_3_+MOG was completely protected and did not show any sign of paralysis. The kinetics of clinical scores and the maximum clinical score in all experimental groups is illustrated in Fig 1A and 1C, respectively. Maximum clinical score determination confirmed that MOG and 1,25(OH)_2_D_3_ alone similarly reduced disease severity whereas their association completely avoided any clinical disease manifestation. EAE incidence is depicted in Table 1 and clearly indicates that treatment with 1,25(OH)_2_D_3_ or MOG were also able to reduce disease incidence. However, their combination reduced disease incidence to zero. As expected, mice from the positive control group lost body weight during the acute disease phase. Mice treated with MOG did not lost weight whereas the ones that received 1,25(OH)_2_D_3_ alone or combined with MOG, lost weight even before paralysis manifestations (Fig 1B).

**Table 1:** EAE incidence in mice treated with 1,25(OH)_2_D_3_+MOG.

Groups	Incidence	p
**EAE**	12/12 (100%)	≤0.001*
**EAE + MOG**	5/12 (42%)	
**EAE + vit D**	3/12 (25%)	
**EAE/ vit D + MOG**	0/12 (0%)	

*Chi-square was performed for EAE incidence. p≤0.001 compared EAE with EAE+MOG, EAE+vitD and EAE/vitD+MOG groups. Data are representative of two independent experiments.

### Quantification of DCs in the spleen

**Fig 2:**
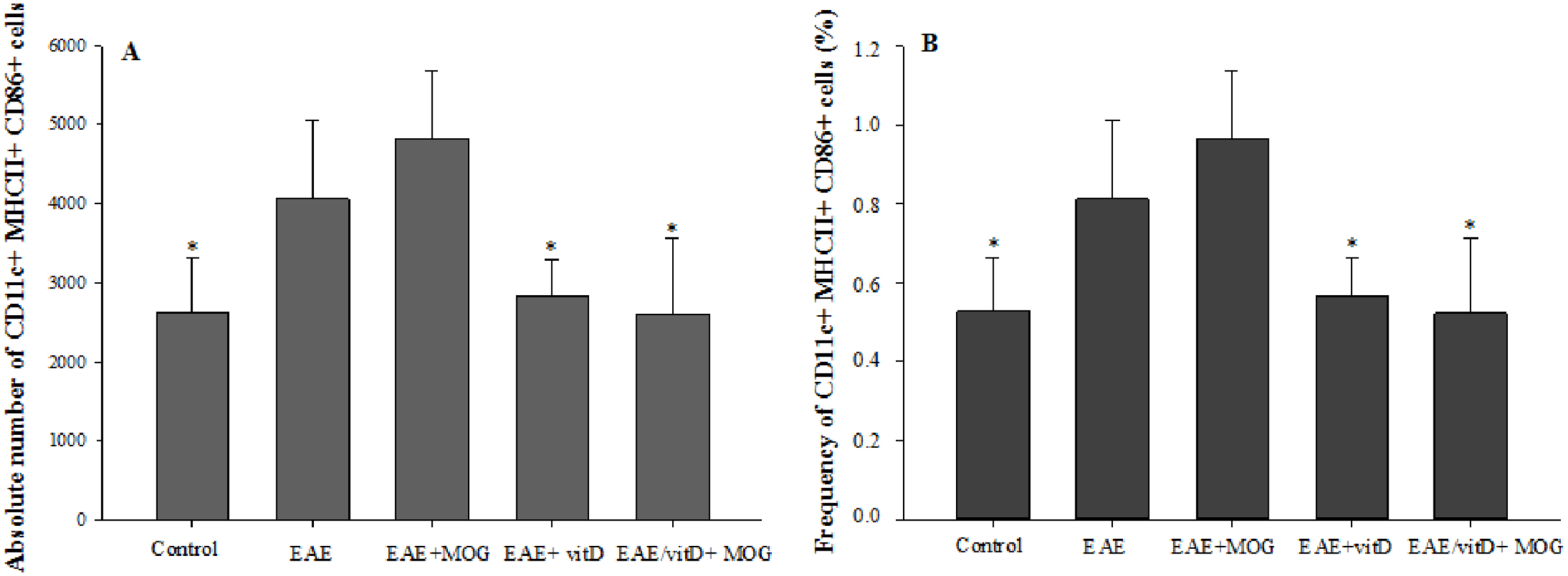
Quantification of splenic dendritic cells in C57BL/6 mice treated with 1,25(OH)_2_D_3_+MOG. Absolute number (**A**) and percentage (**B**) of CD11c+MHCII+CD86+ cells in 500,000 acquired events. Comparisons between groups were made by one way ANOVA followed by Tukey’s test. Results are expressed as mean ± SD of 5 animals per group. * p<0.05 compared with EAE and EAE+MOG group. Data are representative of two independent experiments.

As depicted in Fig 2A, there was a significant increase in the absolute number of mature DCs in the EAE group compared with normal control. Contrarily, there was a significant decrease in the number of mature DCs in the groups treated with 1,25(OH)_2_D_3_ or 1,25(OH)_2_D_3_+MOG compared with EAE and EAE/MOG groups. The same profile was observed in the percentage of these cells among the groups (Fig 2B).

### Cytokine evaluation in the spleen and CNS

**Fig 3:**
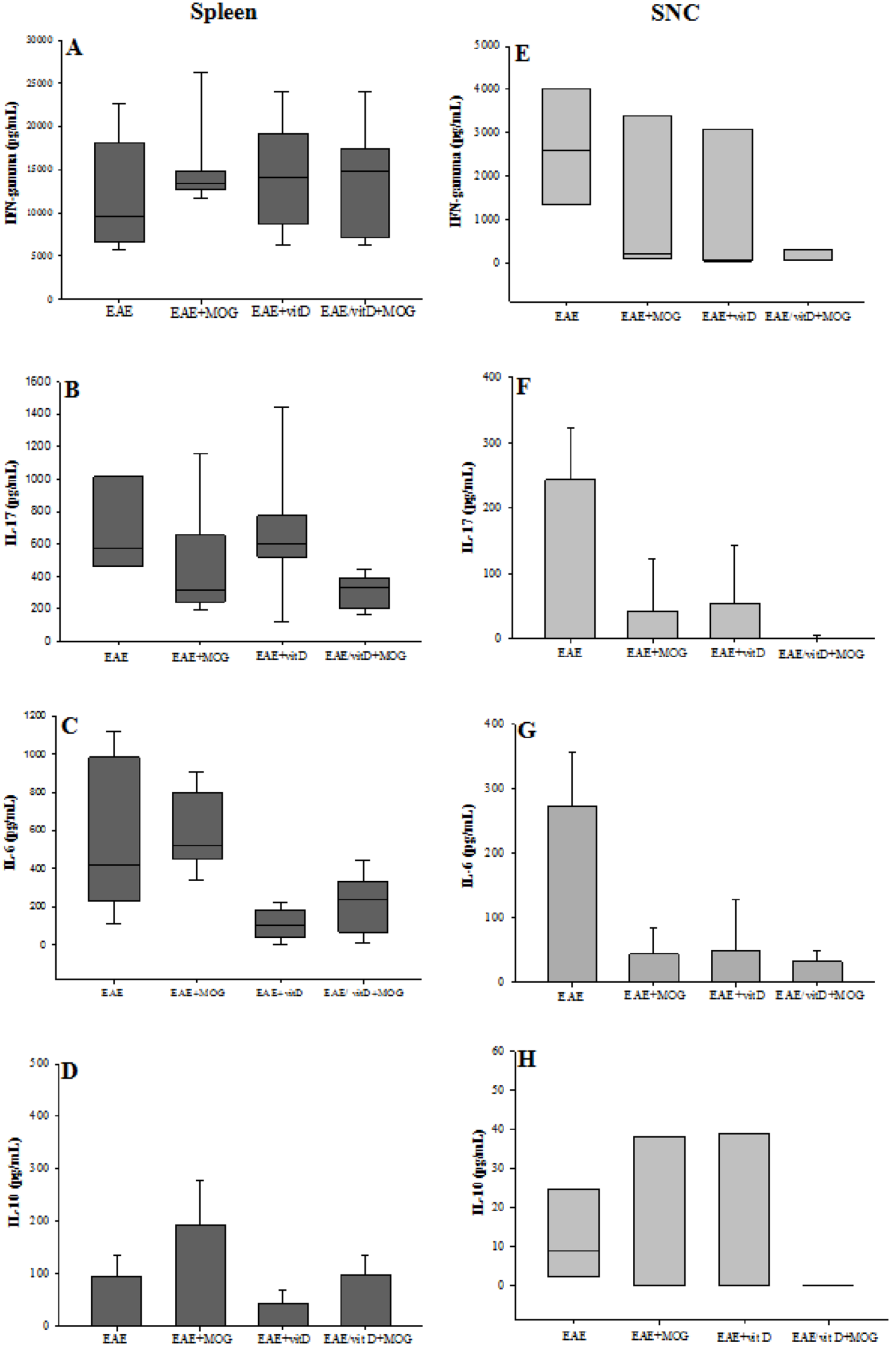
Cytokine production by spleen and CNS cell cultures. IFN-γ (**A** and **E**), IL-17 (**B** and **F**), IL-6 (**C** and **G**) and IL-10 (**D** and **H**) levels were measured in spleen and CNS cell cultures stimulated with MOG. Comparisons between groups were made by one way ANOVA followed by Tukey’s test for parametric variables (**D, F** and **G**) and by Kruskal-Wallis followed by Dunn’s test for non-parametric variables (**A**, **B, C, E** and **H**). Data were presented by mean ± SE or medians (25–75% ranges) of 9 animals per group in spleen cultures or 4 pools (each pool contains cells from brain and spinal cord of 3 mice) per group in CNS cultures. * p<0.05. Data are representative of two independent experiments.

The recall response was evaluated with cell cultures from the spleen and the CNS stimulated with MOG. Similar amounts of IFN-γ were produced by all experimental groups (Fig 3A). IL-17 production was statistically down-modulated in the EAE/1,25(OH)_2_D_3_+MOG compared with the EAE group (Fig 3B). IL-6 was down-modulated in this and also in the EAE/1,25(OH)_2_D_3_ group (Fig 3C). The levels of IL-10 were similar in EAE, EAE/1,25(OH)_2_D_3_ and EAE/1,25(OH)_2_D_3_+MOG groups. However, mice treated only with MOG produced higher levels of IL-10 compared with the three other experimental groups (Fig 3D).

Lower levels of IFN-γ, IL-17, IL-6 and IL-10 were produced by cells eluted from the CNS of treated mice (Fig 3E, 3F, 3G and 3H). However, treatment with 1,25(OH)_2_D_3_+MOG determined the most striking modulation, significantly reducing the production of IFN-γ, IL-17 and IL-6 as shown in Fig 3E, 3F and 3G, respectively.

### Quantification of regulatory T cells in peripheral lymphoid organs

**Fig 4:**
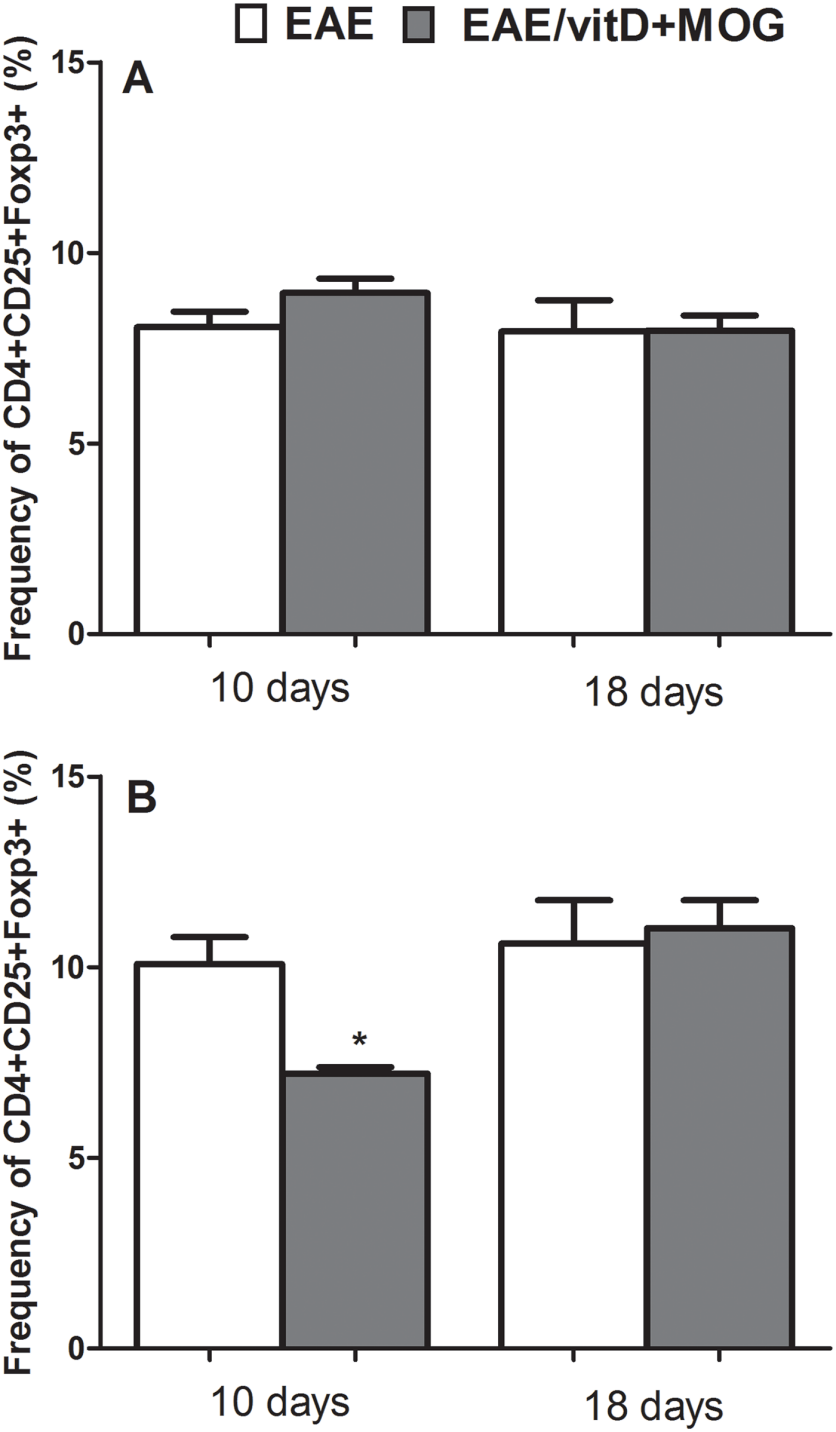
Quantification of regulatory T cells in peripheral lymphoid organs from C57BL/6 mice treated with 1,25(OH)_2_D_3_+MOG. Percentage of CD4+CD25+Foxp3+ T cells in total CD4+ T cell. Spleen (**A**) and lymph nodes (**B**) were collected 10 and 18 days after EAE induction. Analyses were performed in 100,000 acquired events. Comparisons between groups were made by t-test followed by Tukey’s test for parametric variables. Results were expressed as mean of 6–9 animals per group. Data are representative of two independent experiments.

The frequency of Foxp3+ Treg cells was evaluated at the pre-clinical and clinical phases, that is, 10 and 18 days after EAE induction respectively. No differences were observed in the spleen (Fig 4A). However, the frequency of CD4+CD25+Foxp3+ T cells was significantly reduced in the lymph nodes of mice treated with 1,25(OH)_2_D_3_+MOG compared with the EAE untreated group (Fig 4B).

### Inflammatory process in the CNS

**Fig 5:**
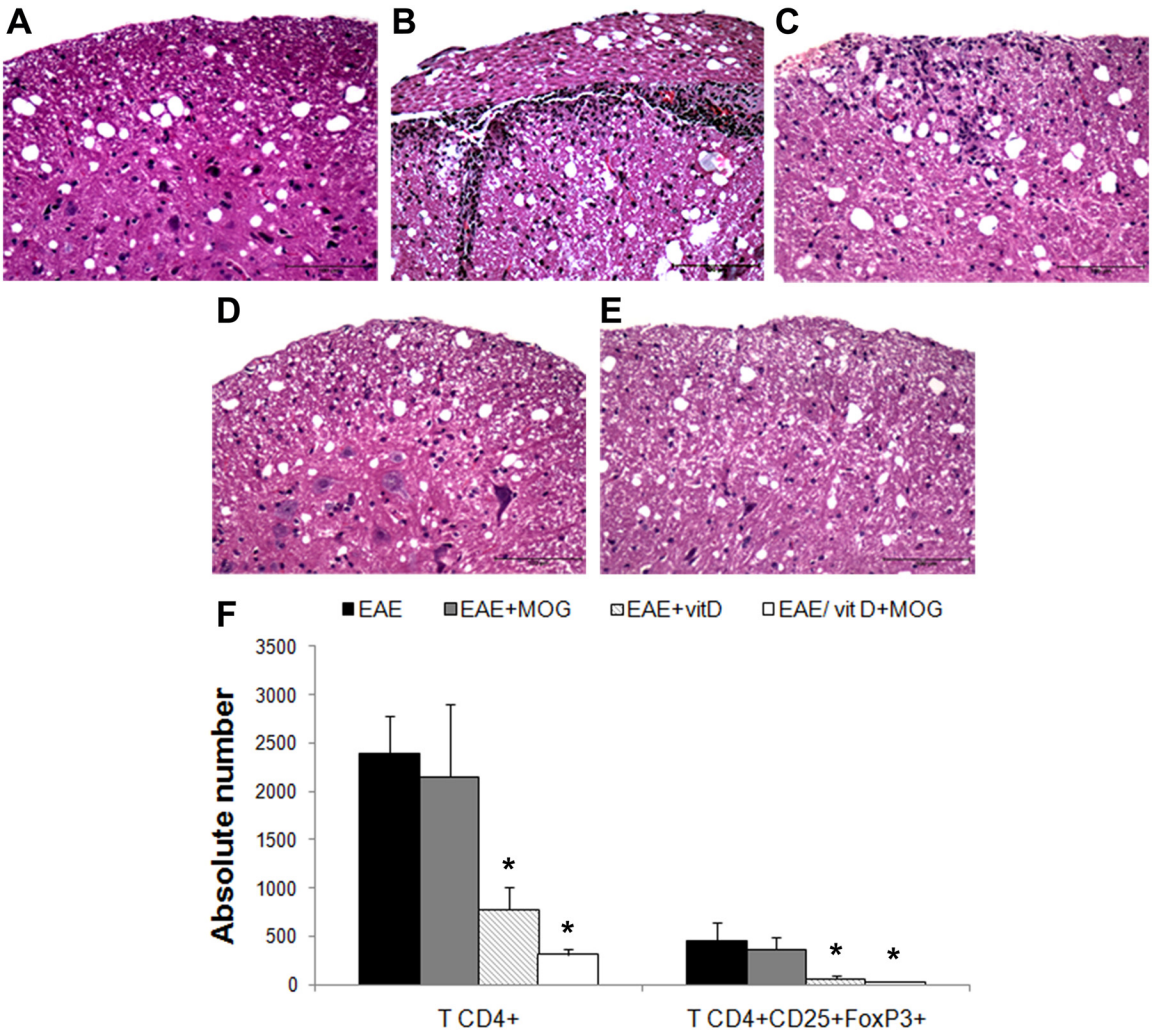
Infiltrating cells in the CNS after treatment of 1,25(OH)_2_D_3_+MOG. Inflammatory process in lumbar spinal cord sections stained with H&E in control (**A**), EAE (**B**), EAE+MOG (**C)**, EAE+vitD (**D**) and EAE/vitD+MOG (**E**) groups and absolute number of CD4+ T cells and CD4+CD25+Foxp3+ T cells in CNS-mononuclear cells (**F**). Regulatory T cells were evaluated in the total CD4+ T cell population and analyses were performed in 20,000 acquired events. Comparisons between groups were made by one way ANOVA followed by Tukey’s test. Data were presented by mean ± SE of 4 pools (each pool contains cells from brain and spinal cord of 3 mice) per group in CNS cultures cells. * p<0.05 in compared with EAE and EAE+MOG group. Scale bar = 100μm. Micrographs are representative of 5 animals per group. Data are representative of two independent experiments.

As expected, the EAE control group showed typical inflammatory foci, with a clear predominance of mononuclear cells in the lumbar spinal cord (Fig 5B). A similar picture was observed in sections from the EAE+MOG group (Fig 5C). Otherwise, samples from EAE+1,25(OH)_2_D_3_ (Fig 5D) and EAE/1,25(OH)_2_D_3_+MOG (Fig 5E) groups showed no inflammation. The absence of inflammation in normal mice is illustrated in Fig 5A. Similar results were observed in brain analysis (not shown). Inflammation was also assessed by CD4+ and CD4+CD25+Foxp3+ T cells quantification in cells eluted from the CNS. The total amount of CD4+ T cells was similar in EAE and EAE/MOG groups. However, a striking reduction was detected in the EAE/1,25(OH)_2_D_3_ and EAE/1,25(OH)_2_D_3_+MOG groups. A similar profile was found when CD4+CD25+Foxp3+ T cells were quantified. In both evaluations, a statistical comparison between EAE/1,25(OH)_2_D_3_ and EAE/1,25(OH)_2_D_3_+MOG did not indicate statistical significance even though down-modulation by the associated treatment was apparently more efficient (Fig 5F).

### Serum levels of calcium and phosphorus

**Fig 6:**
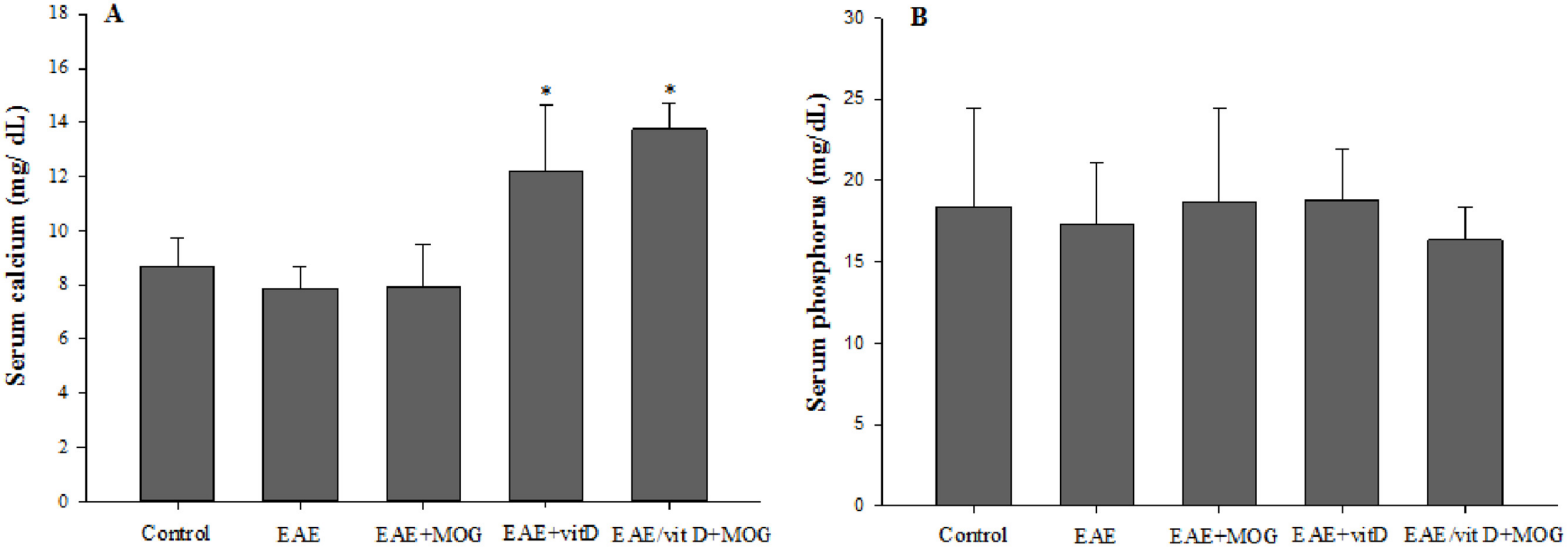
Effect of 1,25(OH)_2_D_3_ (alone or combined with MOG) treatment in calcium and phosphorus levels. Serum calcium (**A**) and phosphorus (**B**) levels were quantified 18 days after EAE induction. Comparisons between groups were made by one way ANOVA followed by Tukey’s test. Data were presented by mean ± SE of 4–6 animals per group. * p<0.05 compared with Control, EAE, and EAE+MOG group. Data are representative of two independent experiments.

1,25(OH)_2_D_3_ treated groups presented significantly higher serum levels of calcium (Fig 6A) but not of phosphorus (Fig 6B) in the moment of euthanasia.

## Discussion

Considering the immunomodulatory properties of active vitamin D and the epidemiological observations that low serum levels of this vitamin are probably associated with increased MS risk [26], we hypothesized that the association of 1,25(OH)_2_D_3_ with MOG could have a protective effect on EAE. The success of MOG+1,25(OH)_2_D_3_ treatment was clearly demonstrated by clinical parameters, that is, disease incidence and maximum clinical score were zero in these animals. However, MOG and 1,25(OH)_2_D_3_ by themselves were also partially effective, being 1,25(OH)_2_D_3_ more efficient than MOG. The observed efficacy of 1,25(OH)_2_D_3_ administration (75% reduction in incidence and a significant decrease in the maximum clinical score) was expected and supported by recent work with similar experimental models [23]. A variety of strategies employing MOG has also been devised, tested and proved promising. MOG can be used alone by nasal administration [27] and is also effective by delivery as a DNA vaccine [28] or as part of fusion proteins [29]. The strong immunomodulatory activity of this association was clearly revealed by the analysis of cytokine production by spleen cells and also by the quantification of DCs in this organ. A significant reduction was detected in IL-6 and IL-17 levels even though IFN-γ and IL-10 levels were not affected. Reduced IL-17 production is particularly relevant because it is considered a highly encephalitogenic cytokine in EAE and MS patients [30,31]. In the group that received this combined treatment there was a clear reduction in the amount of mature DCs in the spleen. This effect was attributed solely to 1,25(OH)_2_D_3_ because MOG alone increased, even though not substantially, the amount of these cells. Much of the knowledge in this area relates to the *in vitro* effect of 1,25(OH)_2_D_3_ on DCs. In the presence of 1,25(OH)_2_D_3_ these cells differentiate into a phenotype that resembles semi-mature DCs that are poor IL-12 producers and express low amounts of co-stimulatory and MHCII molecules. These 1,25(OH)_2_D_3_-modulated DCs usually induce tolerance characterized by a blockage in Th1 and Th17 differentiation together with recruiting and differentiating regulatory T cells [32,33]. The adoptive transfer of 1,25(OH)_2_D_3_ modified DCs has been considered the most promising immunotherapeutic approach for autoimmune diseases. Our results strongly suggest that a similar immunomodulatory effect can be achieved *in vivo* by the combined treatment with 1,25(OH)_2_D_3_ and the specific antigen.

Unexpectedly, no increase was found in the amount of CD4+CD25+Foxp3+ T cells in spleen and lymph nodes. We even found, at the preclinical stage, a significant decrease in their percentage in the draining lymph nodes. Our interpretation is that protection in this case is independent of Treg induction relying mostly on the concomitant presence of immature DCs and the self-antigen. In this scenario, MOG specific T cells could be deleted by the contact with immature DCs as has been demonstrated in various experimental scenarios [15,34,35]. Even though Treg induction is being predominantly linked to 1,25(OH)_2_D_3_ immunoregulatory effects [36, 37] a few reports also show that active vitamin D is able to directly inhibit the expression of Foxp3 [22].

IL-10 has been considered essential to mediate the protection triggered by active vitamin D in EAE [38]. However, our results did not show increased production of this cytokine. A possible explanation for these apparently contradictory findings could be the 1,25(OH)_2_D_3_ administration route and/or its association with the neuroantigen. Spach et al (2006) [38], used only 1,25(OH)_2_D_3_ by oral route whereas we employed an association of active vitamin D + MOG by intraperitoneal route.

The higher efficacy of 1,25(OH)_2_D_3_+MOG treatment and, to a lesser extension, of 1,25(OH)_2_D_3_ alone, was confirmed when sections from the CNS, local cytokine production, and the characteristics of the CD4+ T cells eluted from this tissue were analyzed. Distinctly from EAE and EAE/MOG groups that presented clear cell infiltrates in the CNS, no inflammation was found in the CNS of 1,25(OH)_2_D_3_+MOG treated animals. However, it was possible to elute cells from the CNS of all experimental groups. One of the major differences was the amount of cells; EAE and EAE/MOG groups had a significantly higher number of CD4+ T cells than EAE/1,25(OH)_2_D_3_ and EAE/1,25(OH)_2_D_3_+MOG groups. In addition, the total number of CD4+CD25+Foxp3+ T cells was significantly lower in the two groups that received 1,25(OH)_2_D_3_.

Altogether, these results strongly suggest that 1,25(OH)_2_D_3_ is working as a tolerogenic adjuvant. The potential usefulness of other immunosuppressants as tolerogenic adjuvants was initially suggested by Kang et al. 2008 [39]. These authors demonstrated that BALB/c mice, with a pre-established delayed type hypersensitivity reaction to OVA, could be desensitized by treatment with an OVA-peptide associated with dexamethasone. The tolerogenic mechanism included a blockage in DCs maturation and also an expansion of CD4+CD25+Foxp3+ regulatory T cells. This drug was also tolerogenic and prophylactic when associated with an insulin peptide in the NOD diabetes model. The efficacy of this concept was also tested in EAE. Kang et al. 2009 [40], used FK506 as a tolerogenic adjuvant combined with a DNA vaccine encoding MOG to prevent EAE development.

Based on the above results we postulate that the combined therapy with specific antigens associated with 1,25(OH)_2_D_3_ is potentially effective to block the development of autoimmune diseases. Our data, that is a new contribution to this field, warrant additional studies to support the potential indication of 1,25(OH)_2_D_3_ + MOG in the clinical setting.

## Supporting Information

S1 File Data used in the analyses of “Treatment with vitamin D/MOG association suppresses experimental autoimmune encephalomyelitis”. (XLSX)
