# Correction: Treatment with Vitamin D/MOG Association Suppresses Experimental Autoimmune Encephalomyelitis

**DOI:** 10.1371/journal.pone.0131260

**Published:** 2015-07-17

**Authors:** The PLOS ONE Staff

There is an error in Fig 3, “Cytokine production by spleen and CNS cell cultures.” The publisher apologizes for this error. Please view Fig 3 here.

**Fig 3:**
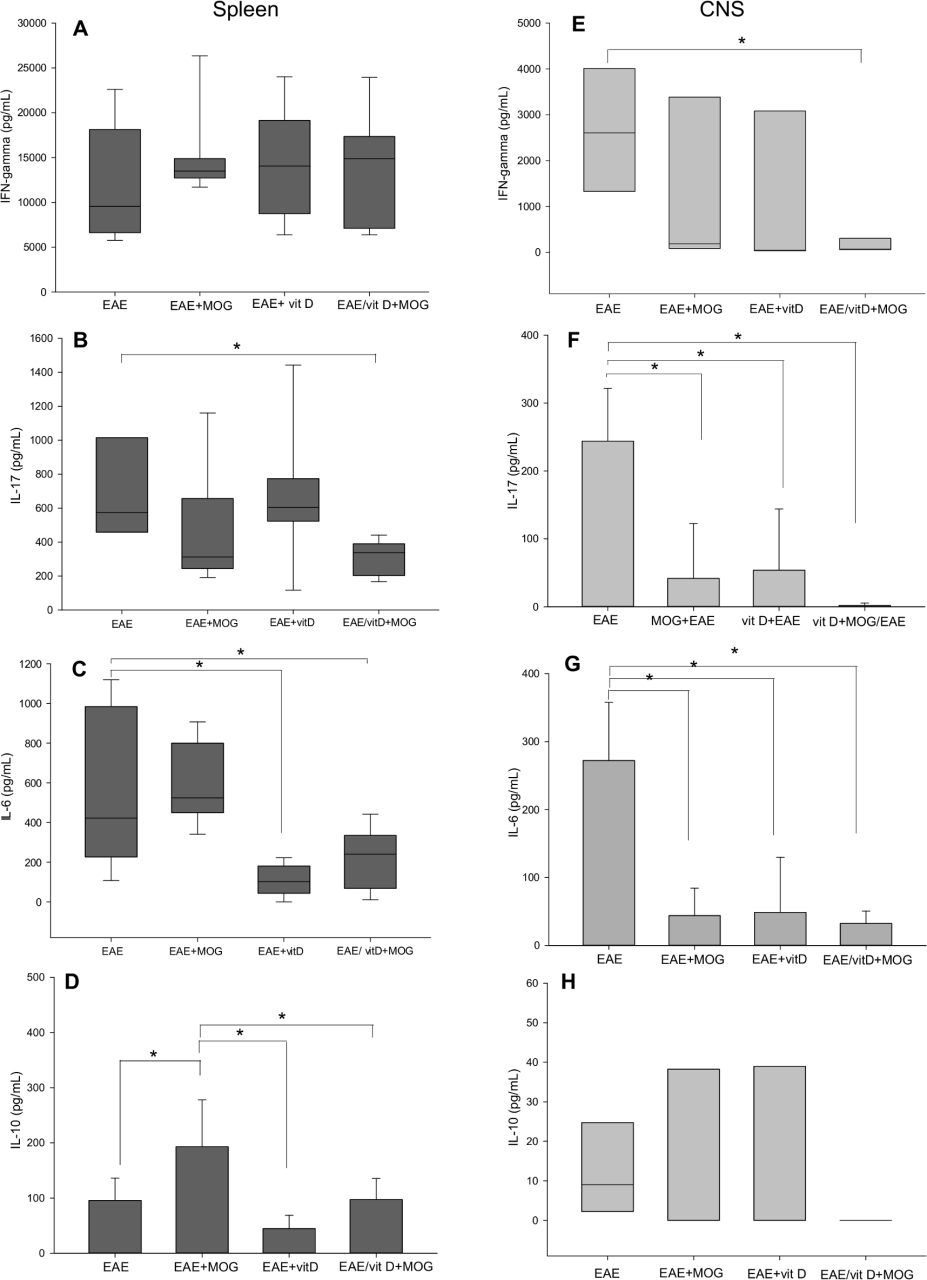
Cytokine production by spleen and CNS cell cultures. IFN-γ (**A** and **E**), IL-17 (**B** and **F**), IL-6 (**C** and **G**) and IL-10 (**D** and **H**) levels were measured in spleen and CNS cell cultures stimulated with MOG. Comparisons between groups were made by one way ANOVA followed by Tukey’s test for parametric variables (**D, F** and **G**) and by Kruskal-Wallis followed by Dunn’s test for non-parametric variables (**A**, **B, C, E** and **H**). Data were presented by mean ± SE or medians (25–75% ranges) of 9 animals per group in spleen cultures or 4 pools (each pool contains cells from brain and spinal cord of 3 mice) per group in CNS cultures. * p<0.05. Data are representative of two independent experiments.
